# Genetic Diversity Analysis of Cotton Cultivars Using a 40K Liquid Chip in Northern Xinjiang

**DOI:** 10.3390/ijms27010545

**Published:** 2026-01-05

**Authors:** Zhihong Zheng, Ningshan Wang, Shangkun Jin, Kewei Ning, Guoli Feng, Haiqiang Gao, Zhanfeng Si, Tianzhen Zhang, Nijiang Ai

**Affiliations:** 1 Shihezi Academy of Agricultural Sciences, Shihezi 832000, China; 2 Modern Seed Industry Research Institute, College of Agriculture and Biotechnology, Zhejiang University, Hangzhou 310058, China

**Keywords:** ZJU CottonSNP40K, Northern Xinjiang cotton varieties, genetic relationship

## Abstract

Genetic diversity and kinship information of cotton germplasm resources are fundamental to breeding, providing a theoretical basis for the rational selection of hybrid parents and further breeding of new varieties with high yield, high quality, and multi-resistance. This study utilized cotton varieties that have been used for variety improvement or are widely planted in the Northern Xinjiang cotton region as materials. Genotyping was performed using the ZJU CottonSNP40K chip to analyze genetic diversity and kinship relationships. A total of 26,852 high-quality SNP markers were obtained, including 15,222 SNPs in subgenome A and 11,630 SNPs in subgenome D. The number of SNPs per chromosome ranged from 547 (A04) to 2168 (A08). Based on phylogenetic tree and principal component analysis, the 83 materials were clustered into 3 major subgroups. Group I contained varieties introduced from the former Soviet Union and the United States, which have become important parents for cotton breeding in Northern Xinjiang. Among them, as many as 27 varieties were derived and selected from the introduced US variety ‘Beiersinuo’ as a parent. While playing an important role in cotton breeding in Northern Xinjiang, this has also led to the current situation where the genetic base of Northern Xinjiang varieties is relatively narrow (average kinship coefficient 0.72). It clarifies the significant role of introduced American variety ‘Beiersinuo’ in the breeding of Northern Xinjiang cultivars and provides theoretical guidance for broadening the genetic base of Northern Xinjiang cotton varieties.

## 1. Introduction

Xinjiang is China’s most important high-quality commercial cotton production base. Based on climatic conditions, the Xinjiang cotton region is generally divided into the Northern Xinjiang early-maturing cotton region and the Southern Xinjiang early-to-medium-maturing cotton region. The Northern Xinjiang early-maturing cotton region holds an extremely important position in Xinjiang’s high-quality cotton production, accounting for 30% of the total planting area and over 32% of the total product in in Xinjiang. The Northern Xinjiang cotton production area boasts superior farmland infrastructure, high mechanization levels, advanced irrigation techniques, and high cultivation management standards, with a favorable market for the cotton industry. Over the years, the “Xinluzao” series varieties have become the dominant cultivars in this region across different periods due to their good early maturity, high yield, and stability, making outstanding contributions to cotton production development in Northern Xinjiang. Analyzing the genetic diversity and kinship relationships of cotton cultivars is of great significance for promoting the breeding of new cotton varieties suitable for the Northern Xinjiang cotton region with early maturity, high yield, superior quality, and multi-resistance.

In-depth research on the genetic diversity and kinship relationships of cotton germplasm resources is the foundation of crop breeding and variety improvement. Through systematic classification and genetic structure analysis of cotton germplasm resources, their genetic variation and gene flow can be effectively evaluated, thereby providing a scientific basis for germplasm innovation and new variety breeding [1,2,3]. Domestic and international scholars have conducted detailed research on cotton variety diversity using morphological traits, isozyme markers, and molecular marker technologies [4,5,6,7,8,9]. Single-nucleotide polymorphism (SNP), the most common form of variation in the genomes of many species with a high distribution rate in the genome, is widely used in genetic map construction, genetic diversity analysis, variety identification, and molecular marker-assisted breeding in crops [1,2,10,11,12,13]. With the continuous development of next-generation sequencing technologies and the gradual refinement of SNP marker technology, corresponding SNP detection techniques have also evolved. Among them, SNP chips, as a detection method integrating advantages such as high throughput, miniaturization, and automation, have been widely applied in population genetics, QTL mapping, and candidate gene screening in various plants [14,15,16,17]. For example, a genetic linkage map, constructed using the GBW16K array-based genotyping of a recombinant inbred line population derived from a cross of the CIMMYT wheat line Yaco “S” and the Chinese landrace Mingxian169, enables the identification of resistant candidate genes [18]. Zhejiang University developed the “ZJU CottonSNP40K” cotton chip that has been widely used in cotton biological breeding [19], for example, in constructing ultra-high-density genetic maps [19] and cotton diversity analysis [20].

This study utilizes the high-coverage “ZJU CottonSNP40K” cotton chip [19] to analyze the genetic diversity of 83 backbone materials for cotton variety improvement and main cultivars in the Northern Xinjiang cotton region. Combined with population genetics methods, it explores their kinship relationships, providing a theoretical basis for the rational selection of hybrid parents.

## 2. Results

### 2.1. The SNP Distribution Characteristics in the Northern Xinjiang Cotton Population

**Figure 1:**
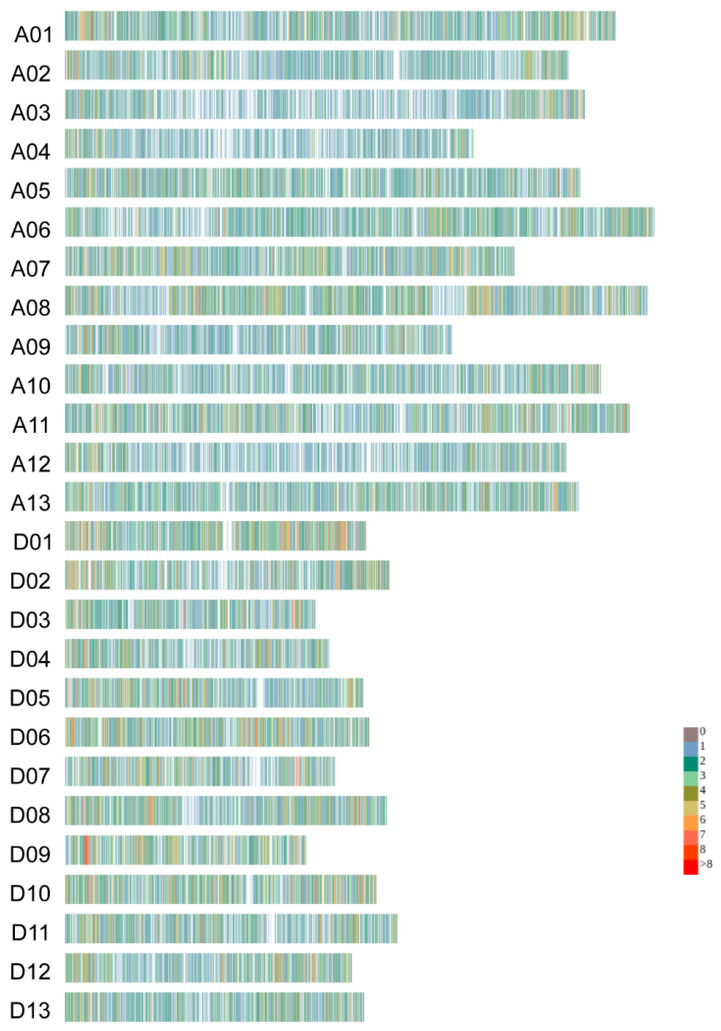
The density of SNPs across the chromosomes. The data is shown in 1 Mb sliding windows.

After filtering, 83 *G. hirsutum* cultivars, including five varieties introduced from the USA and the former Soviet Union (108Φ, 611Б, C1470, KK1543, and Beiersinuo), and 78 cotton varieties from Northern Xinjiang (mainly the Xinluzao series varieties), yielded a total of 26,852 high-quality SNP markers, with A subgenome containing 15,222 SNPs and D subgenome containing 11,630 SNPs. The number of SNPs per chromosome ranged from 547 (A04) to 2168 (A08) (Table 1). The SNP density was 12.4 per Mb, relatively evenly distributed across each chromosome (Table 1, Figure 1). The maximum density was 18.4 per Mb (D09), and the minimum density was 5.8 per Mb (A02).

### 2.2. Genetic Diversity in Northern Xinjiang Cotton Cultivars

**Figure 2:**
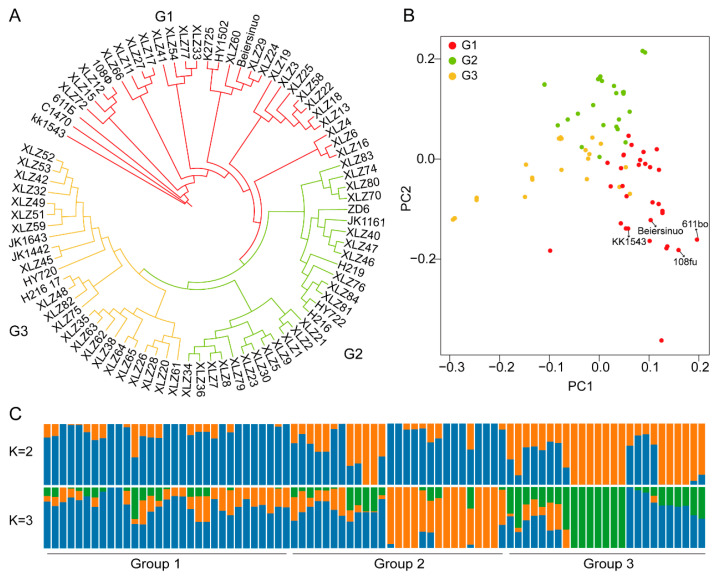
Clustering and population structure of cotton varieties in Northern Xinjiang. (**A**) Phylogenetic tree of early maturing varieties, different colors represent different groups (G1 to G3) (**B**) Principal component analysis. Different colors represent different groups. Arrows indicate important foreign-introduced parental lines. (**C**) Structure analysis with K = 2 and K = 3. The x-axis represents the different accessions. The orders and positions of accessions are consistent with those in the phylogenetic tree when K = 3.

To investigate the genetic relationships among these materials, genetic distances between every two of the 83 materials were calculated using Phylip software to construct a phylogenetic tree. The results showed that these materials could be divided into three subgroups (Figure 2A and Table S1). Group I contained 31 materials: the introduced early-maturing materials from the USSR (108Φ, 611Б, C1470, KK1543), the US early-maturing material Beiersinuo, and varieties primarily bred by units such as the Agricultural Science Institute of the 7th Division, i.e., the first batch of early varieties bred using foreign varieties as foundational parents. Group II contained 27 materials, primarily bred by Shihezi Academy of Agricultural Sciences and Hexin Seed Industry. Group III contained 25 materials, primarily selected and bred by Xinjiang Academy of Agricultural and Reclamation Sciences, Huiyuan Seed Industry, etc. The results of principal component analysis (PCA) and population structure analysis were consistent with the phylogenetic tree results (Figure 2B,C), confirming the accuracy of this classification. Analysis of the kinship matrix revealed that the average kinship coefficient among these 83 materials reached 0.72. Specifically, the average kinship coefficient was 0.72 for Group I, 0.75 for Group II, and 0.77 for Group III. These results indicate that cotton varieties in Northern Xinjiang have a narrow genetic base and low genetic diversity.

### 2.3. Genetic Basis of Northern Xinjiang Cotton Cultivar Improvement

**Figure 3:**
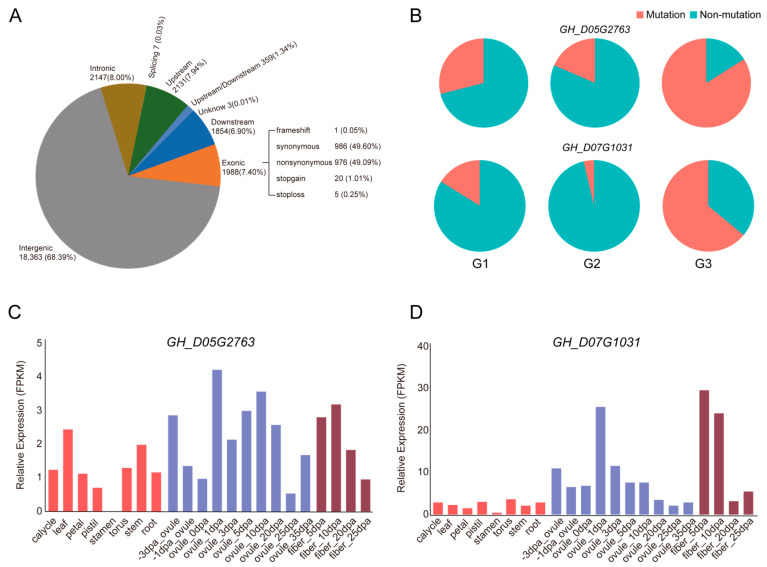
Genetic variation and expression profiles of *GH_D05G2736* and *GH_D07G1031*. (**A**) Statistics of SNP annotation information (**B**) Proportional distribution of SNPs occurred in *GH_D05G2736* and *GH_D07G1031* across different cotton populations. The pie chart shows the percentage of SNPs among G1, G2 and G3 group. (**C**) Expression level of *GH_D05G2736* in various tissues (**D**) Expression level of *GH_D07G1031* in various tissues.

Annotation of these SNPs revealed that 18,363 SNP variants occurred in intergenic regions, 1988 SNPs occurred in exonic regions, 2147 SNPs occurred in introns, 2131 SNPs occurred in gene promoters, and 1854 SNPs occurred in downstream regulatory regions (Figure 3A). This indicated that variants occurring in gene regions and regulatory regions are relatively few, but these sites are highly likely to contribute to trait differences among different varieties. Further filtering of SNPs occurring in exons (filtering steps detailed in methods) yielded 27 non-synonymous mutation type SNPs. These SNPs exhibited significantly different distribution frequencies among the different clustered groups (Table S2), suggesting that these variant sites may represent points of population differentiation, breeding selection, or specific variation. For example, the SNPs carried by *GH_D05G2763* and *GH_D07G1031* both had higher distribution frequencies in Group III (Figure 3B), indicating that these variations arose during later breeding processes. *GH_D05G2763* harbored a non-synonymous mutation (C to T) in the first exon, causing an amino acid change from Leucine to Phenylalanine (Table S2). This gene encodes a Leucine-rich repeat receptor-like protein kinase (LRR-RLK), expressed throughout various developmental stages (Figure 3C). The *GH_D07G1031* gene harbored a non-synonymous mutation (T to A) in an exon, causing an amino acid change from Valine to Glutamic Acid (Figure 3D). This gene also encodes a receptor protein kinase (hercules receptor kinase), highly expressed in 1 DPA at ovules and 5 DPA and 10 DPA at fiber tissues (Figure 3D), suggesting it may play a key role in fiber initiation and elongation. Receptor kinases have been reported to play important roles in regulating plant reproductive growth, immune responses, and abiotic stress responses [21,22,23]. Therefore, these kinase proteins carrying variant sites may contribute to phenotypic variation in Xinjiang cotton varieties.

## 3. Discussion

Molecular marker technology, such as RFLP and SRR, is widely used in previous genetic diversity analysis of cotton varieties [4,6,9]. However, the practical limitations of these molecular markers, including their finite number and operational complexity, restrict their use in large-scale studies. With the publication of the *Gossypium hirsutum* reference genome [24]. SNP is approaching the ultimate standard for variation detection at the molecular level [1,25,26,27]. Due to the high-cost of re-sequencing of large population, efficient and low-cost SNP genotyping technology has become the optimal choice for SNP detection. Currently, low-cost liquid-phase SNP arrays have been developed in multiple species for breeding research [16,18,28,29]. The “ZJU CottonSNP40K” chip [19], developed using SNP-based targeted sequencing genotyping technology based on the cotton genome, was used in this study for genotyping target sites in Northern Xinjiang cotton cultivars. This cotton chip that has been widely used in cotton biological breeding, such as QTL mapping [19,20].

Pedigree analysis indicates that the parental sources of Northern Xinjiang cotton cultivars mainly originate from three varieties: Beiersinuo, KK1543, and 611Б. The first lineage uses Beiersinuo as a parent to breed Xinluzao 6, Xinluzao 16, Xinluzao 27, and Xinluzao 28 through hybridization and selection. Subsequently, using Xinluzao 6, Xinluzao 16, and Xinluzao 28 as backbone parents, a series of varieties were developed. For example, Xinluzao 22 was bred from the elite line 451 and Xinluzao 6. Xinluzao 29 was selected from lines of Xinluzao 16. The second lineage uses KK1543 as a parent, breeding Xinluzao 3 and Xinluzao 4 through multiple rounds of hybridization. The third lineage uses 611Б and Sining SA as parents to breed Xinluzao 1. Shihezi Academy of Agricultural Sciences further used Xinluzao 1 as a backbone parent to breed Xinluzao 2, Xinluzao 8, and Xinluzao 36. These pedigrees provide valuable genetic information and references for parental selection in Northern Xinjiang cotton breeding, aiding in optimizing breeding strategies. This study, based on 40K liquid SNP chip technology, systematically analyzed the genetic diversity and kinship relationships of 83 *G. hirsutum* main cultivars in the Northern Xinjiang cotton region, revealing the characteristics of their genetic structure and the genetic bottleneck in parental utilization. The results indicate that Northern Xinjiang cotton cultivars could be divided into three major genetic groups, corresponding to different parental sources and breeding pedigrees. Group I was mainly composed of varieties introduced from the former Soviet Union and the USA (such as Beiersinuo, KK1543, etc.) and their derivatives. Among them, Beiersinuo, as a core parent, directly or indirectly gave rise to 27 bred cultivars. Its excessive use resulted in a high average kinship coefficient of 0.72 within the population, significantly exacerbating genetic background homogenization. This finding aligns with previous conclusion [1,2,30,31] about the narrow genetic base of Chinese self-bred *G. hirsutum* varieties, further confirming at the molecular level that the singular utilization of exotic germplasm resources is a key factor limiting the genetic diversity of cotton in Northern Xinjiang. In contrast, Groups II and III are mainly composed of varieties bred by local research units in Xinjiang (Figure 2). Their parental sources rely more heavily on early-introduced former Soviet germplasm (e.g., 611Б), but genetic diversity remains at a low level, indicating that the scope of parental selection in current breeding strategies needs to be expanded.

The SNP annotation results showed that only about 7.4% of the variants were located in gene coding or regulatory regions, that are strong candidates for mediating the phenotypic differences observed. Further filtering of SNPs occurring in exons, yielded 27 non-synonymous mutation type SNPs. These SNPs exhibited significantly different distribution frequencies among the different clustered groups. This pattern is consistent with genetic differentiation between populations, which could arise from various processes including genetic drift, direct or indirect selection, or localized mutation. We also found two kinase proteins carrying variant sites that may contribute to phenotypic variation in Xinjiang cotton varieties. This provides important clues for subsequent functional gene mining and molecular marker-assisted breeding.

This study achieved more efficient genotyping through high-density SNP chips, and the classification results highly coincided with the subgroup division reported previously, further validating the profound influence of former Soviet germplasm on Xinjiang cotton breeding. However, this study found that the genetic contribution of Beiersinuo far exceeded expectations. Its derivative varieties accounted for 32.5% of the main cultivars in Northern Xinjiang, higher than the contribution ratio of former Soviet varieties (e.g., KK1543 only derived 2 varieties). This phenomenon highlights the risk in commercial breeding of over-reliance on a single high-yielding parent, which may lead to decreased stress resistance and adaptability. In similar international studies, the 63K cotton SNP chip also revealed similar issues, namely that the genetic base of modern varieties has significantly narrowed due to the heavy use of core parents [8]. Therefore, the findings of this study not only provide implications for breeding in Northern Xinjiang but also offer a reference for the sustainable utilization of global cotton genetic resources.

## 4. Materials and Methods

### 4.1. Materials

Eighty-three *G. hirsutum* varieties were collected from cotton breeding units including the Xinjiang Academy of Agricultural Sciences. Variety names and breeding organization information are shown in Table S1. The varieties were uniformly planted in an artificial climate chamber at 28 °C with a photoperiod of 16 h light/8 h dark. True leaf tissue was sampled separately after cotton seedlings reached the two-true-leaves stage. Genomic DNA was extracted using the modified CTAB method [32].

### 4.2. Genotyping and SNP Analysis

Genotyping was performed using the 40K chip [19], based on liquid probe hybridization targeted genotyping technology (Genotyping By Target Sequencing, GBTS), jointly designed and developed by Zhejiang University and Borui Di Biotechnology Co., Ltd. Genotyping was completed by Borui Di Biotechnology Co., Ltd. Quality-controlled clean reads were aligned to the reference genome TM-1 [33] using the BWA-mem alignment method [34]. SNPs with minor allele frequency (MAF) < 0.05, missing rate > 0.8, or identical to the TM-1 reference genome were removed. Ultimately, 26,852 high-quality SNP markers were determined for subsequent analysis.

### 4.3. SNP Variant Annotation

Based on TM-1 reference genome information and annotation files, ANNOVAR software (Version: 2.1.1) was used to annotate and classify genome-wide SNP variants [35]. Variants were mainly classified into the following types: SNPs located in intergenic regions, upstream regions, downstream regions, intronic regions, and exonic regions. SNPs located in the coding sequence (CDS) region were further categorized as stop gain, stop loss, synonymous mutation, non-synonymous mutation, frameshift deletion, and splicing.

### 4.4. Kinship Analysis

Based on the SNP variant information of this population, a phylogenetic tree was constructed using the neighbor-joining (NJ) method in Phylip software (Version: 3.696) [36]. Population structure analysis was performed using ADMIXTURE software (version 1.3.0, https://dalexander.github.io/admixture/ (accessed on 10 January 2025)). Principal component analysis (PCA) was conducted using GCTA software (Version 1.26.0) [37]. Kinship coefficients were estimated using the SNPRelate (version 1.40.0) [38] package in R. After quality control (excluding samples and SNPs with >20% missing data and imputing remaining missing values), a robust kinship matrix was generated by calculating Identity-by-State (IBS) similarities. To ensure reliable computation, genotypes were managed in a GDS file created via the gdsfmt package, and IBS values were computed using all QC-passed SNPs without linkage disequilibrium pruning. The resulting matrix was used to assess genetic relatedness among all pairwise combinations of individuals.

### 4.5. Transcriptome Analysis

The clean RNA-seq reads were mapped to the reference genome of TM-1 [33] using HISAT2 [39] (v 2.1.0) with default settings. High-quality mapping reads were used to calculate gene expression levels using StringTie [40] (v2.1.4) with parameter settings (--fr -e -G).

### 4.6. Published Data Download

The Illumina RNA-seq data of TM-1 was retrieved from the NCBI Sequence Read Archive (BioProject: PRJNA490626) [33] and the published cotton genome sequences was downloaded from COTTONOMICS database [41] (http://cotton.zju.edu.cn/ (accessed on 10 January 2025)), respectively.

## 5. Conclusions

This study utilized the “ZJU CottonSNP40K” chip to conduct a genome-wide genetic analysis of 83 *G. hirsutum* cultivars from the Northern Xinjiang cotton region, obtaining 26,852 high-quality SNP markers. It systematically elucidated their genetic structure characteristics and kinship relationships. The results show that the tested materials can be divided into three major genetic groups. Group I, centered around the introduced US variety “Beiersinuo” as the core parent, accounted for 32.5% of the derivative varieties (Figure S1). Its excessive use led to genetic homogenization within the population (average kinship coefficient 0.72). Groups II and III are dominated by locally bred varieties; their genetic diversity levels also remain low, with average kinship coefficients of 0.75 and 0.77, respectively. From the perspective of genomic variation patterns, the number of SNPs in subgenome A (15,222) was significantly higher than in subgenome D (11,630), and their distribution was uneven across chromosomes (chromosome A08 had the highest marker density). Only 7.4% of SNPs were located in functional regions, which are strong candidates for mediating the phenotypic differences observed. The root cause of the narrow genetic base of current Northern Xinjiang cotton cultivars lies in the singular utilization of exotic germplasm, especially “Beiersinuo”. Future efforts require a diversified parental selection system, prioritizing the integration of wild or local germplasm with stress resistance and high-quality traits, and breaking genetic bottlenecks through molecular marker-assisted selection. This study provides a molecular basis for the efficient utilization of genetic resources in Northern Xinjiang cotton and holds significant guiding importance for promoting the breeding of new varieties with high yield, superior quality, and multi-resistance.

## Figures and Tables

**Table 1:** Distribution of SNPs across different chromosomes.

Chr	Length (bp)	SNP Number	Chr	Length (bp)	SNP Number
A01	118,174,371	1610	D01	64,698,102	1118
A02	108,272,889	625	D02	69,777,850	1003
A03	111,586,618	792	D03	53,896,199	785
A04	87,703,368	547	D04	56,935,404	555
A05	110,845,161	1123	D05	63,929,679	890
A06	126,488,190	1323	D06	65,459,843	1207
A07	96,598,283	1625	D07	58,417,686	837
A08	125,056,055	2168	D08	69,080,421	1052
A09	83,216,487	985	D09	52,000,373	959
A10	115,096,118	947	D10	66,881,427	877
A11	121,376,521	1372	D11	71,358,197	754
A12	107,588,319	867	D12	61,693,100	852
A13	110,367,549	1238	D13	64,447,585	741

## Data Availability

The datasets presented in this study can be found in the figshare database (https://doi.org/10.6084/m9.figshare.30817382 (accessed on 9 December 2025)).

## Supplementary Materials

The following supporting information can be downloaded at: https://www.mdpi.com/article/10.3390/ijms27010545/s1. References [42,43] are cited in the Supplementary Materials.
