# Supplementary figures and images for: Genetic Diversity Analysis of Cotton Cultivars Using a 40K Liquid Chip in Northern Xinjiang
Source: Int J Mol Sci. 2026 Jan 5;27(1):545. doi: 10.3390/ijms27010545 (PMC12787184; doi:10.3390/ijms27010545)

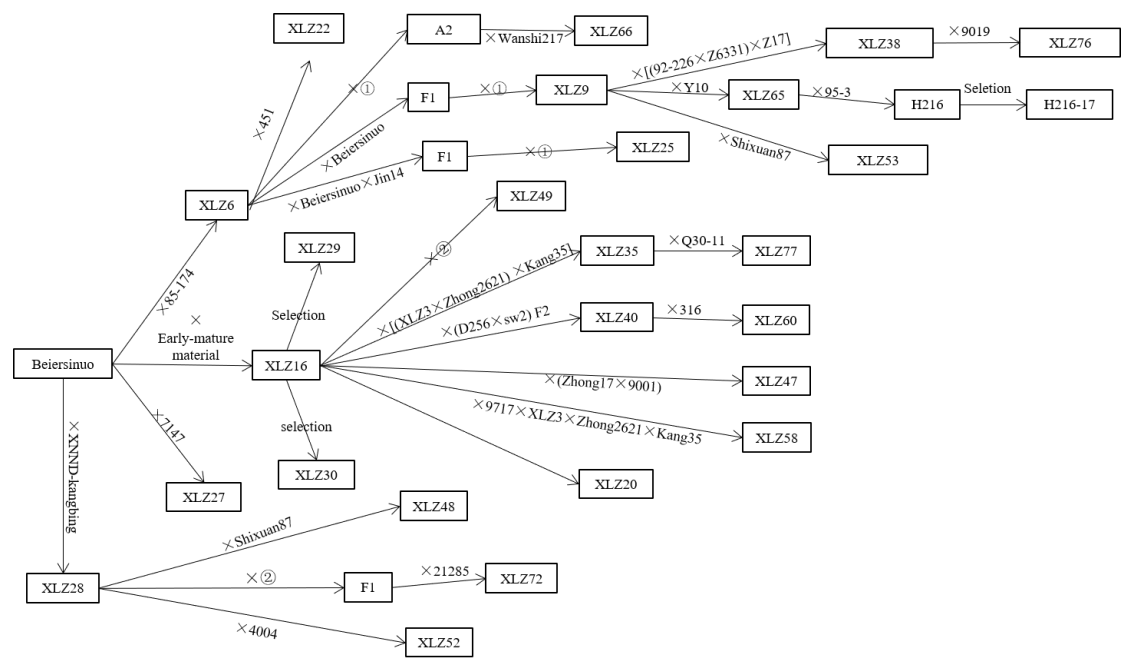

Supplementary Figure S1 Pedigree chart of cotton breeding in Northern Xinjiang.

Supplement: Supplementary file 1 [file ijms-27-00545-s001.zip › Supplementary Figure.pdf]
